# Induction of Interferon-Stimulated Genes by *Chlamydia pneumoniae* in Fibroblasts Is Mediated by Intracellular Nucleotide-Sensing Receptors

**DOI:** 10.1371/journal.pone.0010005

**Published:** 2010-04-06

**Authors:** Srikanth Chiliveru, Svend Birkelund, Søren R. Paludan

**Affiliations:** Department of Medical Microbiology and Immunology, Aarhus University, Aarhus, Denmark; Charité-Universitätsmedizin Berlin, Germany

## Abstract

**Background:**

Recognition of microorganisms by the innate immune system is mediated by pattern recognition receptors, including Toll-like receptors and cytoplasmic RIG-I-like receptors. *Chlamydia*, which include several human pathogenic species, are obligate intracellular gram-negative bacteria that replicate in cytoplasmic vacuoles. The infection triggers a host response contributing to both bacterial clearance and tissue damage. For instance, type I interferons (IFN)s have been demonstrated to exacerbate the course of Chlamydial lung infections in mice.

**Methods/Principal Findings:**

Here we show that *Chlamydia pneumoniae* induces expression of IFN-stimulated genes (ISG)s dependent on recognition by nucleotide-sensing Toll-like receptors and RIG-I-like receptors, localized in endosomes and the cytoplasm, respectively. The ISG response was induced with a delayed kinetics, compared to virus infections, and was dependent on bacterial replication and the bacterial type III secretion system (T3SS).

**Conclusions/Significance:**

Activation of the IFN response during *C. pneumoniae* infection is mediated by intracellular nucleotide-sensing PRRs, which operate through a mechanism dependent on the bacterial T3SS. Strategies to inhibit the chlamydial T3SS may be used to limit the detrimental effects of the type I IFN system in the host response to Chlamydia infection.

## Introduction

*Chlamydia* are gram negative obligate intracellular bacteria and among the smallest bacteria known [1], [2]. *Chlamydia pnuemoniae* causes respiratory tract infections like pneumonia, pharyngitis, and sinusitis. All chlamydial species share a unique biphasic developmental cycle in which the chlamydiae alternate between two morphologically distinct forms. The elementary body (EB) is the infectious but metabolically inert form whereas the reticulate body (RB) is the non-infectious but metabolically active form [3].

Sensing of microbial infections via specific innate immune receptors plays a pivotal role in the proper functioning of the host innate immune system. The germline-encoded recognition receptors (PRR)s act as a molecular switch triggering the innate immune activation and in turn tightly regulate the downstream immune responses to microbial infections [4]. The microbial structures recognized by PRRs are termed pathogen-associated molecular patterns (PAMP)s, and include lipid-rich structures (e.g. lipopolysaccharide) and nucleotides (e.g. RNA). Two important classes of PRRs are the membrane-bound Toll-like receptors (TLR)s, which are localized in the plasma membrane and endosomal compartments, and the Retinoid acid induced gene (RIG)-I-like receptors (RLR)s, which are localized in the cytoplasm [5]. While TLRs recognize a wide range of different structures, the RLRs uniquely recognize RNAs [5].

Intracellular pathogens propagate inside host cells due to dependence on cellular factors for completion of the life cycle. In addition to viruses, certain bacteria and protozoa reside inside cells. *Chlamydia* replicates in cytoplasmic vacuoles in the host cells [2], [3], which triggers an inflammatory host response, contributing to both clearing of the infection and tissue damage [6]. Both in vitro and in vivo studies on *C. pneumoniae* revealed the predominant role of TLR2 over TLR4 following infection [7]. Recent studies reported that in addition to the TLR pathway yet another recognition system involving the cytoplasmic PRRs NOD1 and NOD2 is vital for recognition and clearance of *C. pneumoniae* [8], [9].

Pathogen recognition activates a intracellular signaling programs [5]. One of these leads to activation of the transcription factor interferon (IFN) regulatory factor (IRF)3, which plays a critical role in transcriptional activity of Type I IFN genes and IFN-stimulated genes (ISG)s. IRF3 is expressed constitutively and resides in the cytosol in a latent form [5]. When receiving the appropriate signal, IRF-3 undergoes phosphorylation, dimerization and nuclear translocation. TANK binding kinase (TBK) 1 and IκB kinase (IKK)ε are the essential serine/threonine kinases for phosphorylation of IRF3 and IRF7 [10], [11]. Bacterial pathogens replicating in the cytoplasm of the host cell (e.g. *Listera monocytogenes*, *Legionella pneumophilla*) or releasing bacterial material into the host cytoplasm (group B streptococcus) have recently been reported to induce expression of IFNs and ISGs via cytoplasmic PRRs and the TBK1/IRF-3 axis [12]–[14]. Thus, interaction between bacterial and host cell cytoplasm seems to be sensed and to trigger expression of IFNs and ISGs.

The type 3 secretion system (T3SS) is primarily characterised by the translocation of the bacterial “effectors” into the eukaryotic cell for the manifestation of pathogenesis. The T3SS is important for many gram-negative bacterial species like *Shigella, Salmonella* and *Yerisinia* in delivering the effector proteins into the eukaryotic cells directly [15]. Chlamydia uses a T3SS through the developmental cycle and may possibly employ this to translocate the different effectors into the host cell depending on the phase of the bacterial developmental cycle [16]. More recently, it has been demonstrated that T3SS may also play a part in triggering the innate immune response to infections with different bacteria including *C. pneumoniae* [17], [18].

While Type I IFNs and expression of ISGs has classically been known to stimulate antiviral activity, the recent appreciation of the IFN response in bacterial infections has led to increasing attention on their role in the anti-bacterial defense [19]–[22]. Although the IFN response plays a protective role during some bacterial infections, studies in murine models have suggested that IFNs play a deleterious role in defense against Chlamydia infections. Therefore, knowledge on the mechanisms governing expression of ISGs during infection with *C. pneumoniae* may provide important evidence on the pathology of *Chlamydia* infections. In this work we demonstrate that expression of ISGs during infection with *C. pneumoniae* is mediated by nucleotide-sensing PRRs residing in endosomes and the cytoplasm, and this response is induced through signalling by TBK1/IKKε and is dependent on bacterial replication and the bacterial type III secretion system.

## Materials and Methods

### Cells and infection

Murine embryonic fibroblasts (MEF) cell lines were used, C57BL/6 and mavs^−/−^ were obtained from Z.J.Chen, Department of Molecular Biology, University of Texas, Dallas,TX,USA. myd88^−/−^trif^−/−^ and tbk1^−/−^ ikke^−/−^ were obtained from S. Akira, Department of Host Defense, Research Institute for Microbial Diseases, Osaka University Japan and atg +/− and atg5−/− cells were procured from RIKEN Bio Resource Center, Japan. All cell types were grown in RPMI 1640 (Gibco BRL, Grand Island, NY, USA) containing 25 mM HEPES, 15% (w/v) FCS, 0.3%,10 µg/ml gentamicin in growth medium and gentamicin 2 µg/ml in infection medium at 37°C in presence of 5% CO_2_. Cells were infected with *Chlamydia pneumoniae* strain TWAR CWL029 obtained from American Type Culture Collection (Rockville, MD, USA) at MOI 5 by centrifugation for 30 minutes, 2400 rpm, at 35°C.

### Reagents

Cycloheximide (Sigma Aldrich), a eukaryotic protein biosynthesis inhibitor was used at a concentration of 1 µg/ml. The LPS/TLR4 inhibitor Polymyxin B was purchased from Invivogen and used at a working concentration of 50 µg/ml. The endosomal TLR inhibitor chloroquine (used at a concentration of 10 µM) was obtained from InvivoGen. Ampicillin was used at a concentration of 20 µg/ml. The RNA polymerase III inhibitor ML-60218 (Calbiochem) was used at a concentration of 30 µM and the T3SS inhibitor INP0341, a derivative of salicylidene acylhydrazine and its analogue control INP0406, were kindly donated by Innate Pharmaceuticals, Umeå, Sweden, and used at a concentration of 60 µM.

### Immuno-fluorescence and Microscopy

To analyse chlamydial inclusions and development inside the fibroblasts, cells were cultivated on glass cover slips and infected with 5 culture forming units (IFU) per cell of *C. pneumoniae* were fixed at 36 hr time post challenge. Infected cells were treated with methanol fixed and stained with MAb 15.1 against *Chlamydia* LPS ([23], [24]).

### Real-time PCR (qRT-PCR)

ISG expression at different points in time post infection of *C. pneumoniae* was quantified by quantitative real-time PCR (qRT-PCR) analysis of the generated cDNAs. Total RNAs were then isolated using the High Pure RNA Isolation Kit Protocol (Roche) and subjected to RT-PCR to generate cDNA using p(dT)15 primer (Roche) and Expand Reverse Transcriptase (Roche), allowing reverse transcription of total sample mRNA. The real time PCR was performed using specific primers for murine ISG56 forward primer 5′-ACC ATG GGA GAG AAT GCT GAT-3′, reverse primer: 5′-GCC AGG AGG TTG TGC-3′; murine CCL5 forward primer 5′- ACT CCC TGC TGC TTT GCC TAC-3′, reverse primer 5′- GAG GTT CCT TCG AGT GAC A-3′; murine CXCL10 forward primer 5′- CGA TGA CGG GCC AGT GAG AAT G-3′, reverse primer 5′- TCA ACA CGT GGG CAG GAT AGG CT-3′; murine β-actin forward primer: 5′- TAG CAC CAT GAA GAT CAA GAT -3′, reverse primer: 5′-CCG ATC CAC ACA GAG TAC TT -3′ and quantified using QuantiTect SYBR Green PCR Kit (Qiagen) and the cycle number at detection threshold (crossing point (cp)) weas determined using the Lightcycler (Roche) analyzer and the LightCycler Software 3.0.

### Statistics

The data are presented as means ± SD. The statistical significance was estimated with the Wilcoxon rank sum test (*p*-values of <0.05 were considered to be statistically significant).

### Reproducibility of data

The results shown in this work are derived from data that are representative for the results obtained. For each series of experiments, two to five independent repetitions were performed.

## Results

### *Chlamydia pneumoniae* induces expression of ISGs with a delayed kinetics compared to viruses

**Figure 1:**
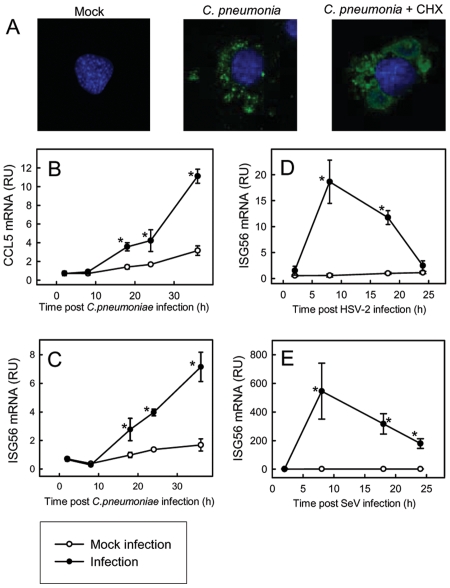
*Chlamydia pneumoniae* induces expression of ISGs with a delayed kinetics compared to viruses. (A) MEF cells were infected with *C. pneumoniae* (5 UFU/cell) in presence or absence of cycloheximide (CHX, 1 µg/ml). 36 hrs post infection, the cells were fixed and stained with an antibody against chlamydial LPS and fluorescence/Nomaski images were obtained. (B–E) MEFs were infected with (B–C) *C. pneumonia*, MOI 5(D) HSV-2, MOI 1, or (E) SeV, MOI 1 for the indicated time intervals. Total RNA was harvested and mRNA levels of ISG56 and CCL5 were determined by real-time PCR and normalized to β-actin. The data are presented as means of triplicate cultures +/− st.dev. Similar results were obtained in 2–5 independent experiments. RU, relative units. Infection-induced gene expression significantly above the expression induced by mock infection at the same tine point (p<0.05) is marked with an asterisk, *.

In order to test whether *C. pneumoniae* establishes productive infection in murine fibroblasts, we treated the cells with the bacteria for 36 h and looked for intracellular vacuoles by confocal microscopy. As shown in Fig. 1A, *C*. *pneumoniae* infection did lead to development of small bacterial inclusions, and this was strongly enhanced if the cells were treated with the eukaryote protein synthesis inhibitor, cyclohexamide. This shows that the cell can mount a defence against the infection. All the following experiments were done without cyclohexamide treatment. To evaluate the expression of ISGs in response to *C. pneumoniae* infection, we harvested total RNA from cells receiving bacterial or mock infection for the indicated time intervals and looked for expression of CCL5, ISG56, and CXCL10. All three ISGs were induced by *C. pneumoniae* infection, with a slow kinetics, where the mRNA levels did not exceede background levels for the first 8 h post bacterial challenge and subsequently were increasingly steady through the 36 h of the experiment (Fig. 1B–C and data not shown). For comparison, we examined the expression of ISG56 in response to two viruses, namely herpes simplex virus (HSV, a DNA virus) and Sendai virus (SeV, an RNA virus). Interestingly, while both viruses induced a potent ISG56 response, this occurred through a rapid and transient kinetics (Fig. 1D–E). Thus, *C. pneumoniae* induces expression of ISGs with a delayed kinetics compared to viruses.

### *Chlamydia pneumoniae*-induced expression of ISGs is dependent on bacterial recognition by endosomal and cytoplasmic nucleotide-sensing PRRs

**Figure 2:**
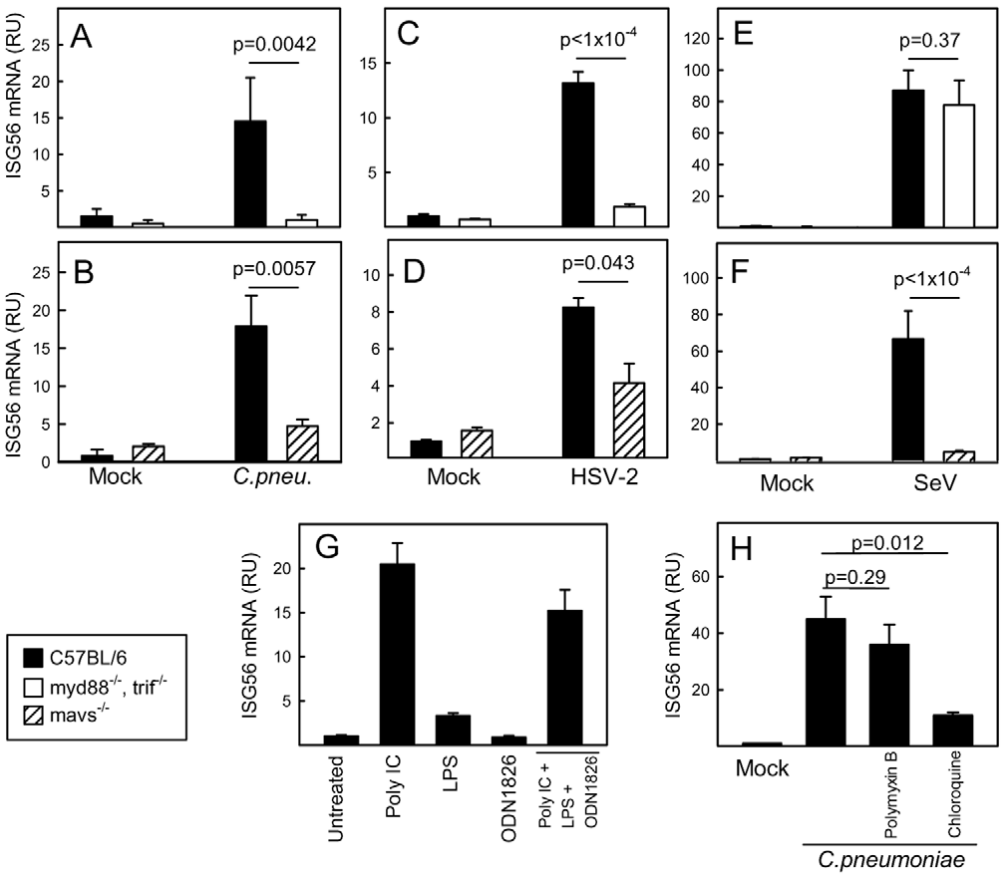
*Chlamydia pneumoniae*-induced expression of ISGs is dependent on bacterial recognition by endosomal and cytoplasmic nucleotide-sensing PRRs. (A–F) WT, myd88^−/−^ trif^−/−^ and mavs^−/−^ MEFs were infected with (A–B) *C. pneumoniae*, MOI 5, for 36 h (C–D) HSV-2, MOI 1, 6 hrs, or (E–F) SeV, MOI 1, 6 hrs. Total RNA was harvested and levels of ISG56 mRNA were determined by real-time PCR. (G) MEFs were stimulated with polyIC (25 µg/ml), LPS (200 ng/ml), and ODN1826 (1 µM) alone or in combination as indicated. Total RNA was harvested 6 hrs post-stimulation, and levels of ISG56 mRNA were determined by real-time PCR. (H) The cells were infected with *C. pneumonia*, MOI 5, for 18 h in the presence or absence of polymyxin B (50 µg/ml) or chloroquine (10 µM) before harvest of total mRNA. The measured ISG56 mRNA levels were normalized to β-actin, and data are presented as means of triplicate cultures +/− st.dev. Similar results were obtained in 2–3 independent experiments. RU, relative units.

Recent evidence suggests that induction of type I IFN and ISG expression by bacteria is mediated mainly through intracellular PRRs, with RNA and DNA constituting important PAMPs [12], [14], [25]–[27]. To address the role of TLRs and RLRs in stimulation of the IFN response during infection with *C. pneumoniae*, we infected wildtype (WT), myd88^−/−^trif^−/−^ and mavs^−/−^ MEFs with the bacteria and measured accumulation of ISG56 mRNA 18 h post infection. Interestingly, the ability of *C. pneumonia* to trigger the IFN response was dependent on intact pathways from both TLRs (MyD88, TRIF) and RLRs (MAVS) (Fig. 2A and 2B). Both knock-out cells stimulated normal induction of ISGs in response to transfected virus-derived DNA (data not shown). For the viruses, which also induced expression of ISG56, HSV-2 relied on both TLRs and RLRs (Fig. 2C and 2D) whereas SeV triggered the response entirely via RLRs (Fig. 2E and 2F).

To investigate which PRRs may be involved in innate recognition of *C. pneumonia*, we treated MEFs with PAMPs, known to activate IFN-inducing TLRs and RLRs, and measured ISG56 mRNA expression. The TLR3 agonist polyIC strongly induced ISG56 expression, and the TLR4 agonist LPS induced a modest but significant response (Fig. 2G). Intracellular delivery of polyIC, which activates RLRs [28], also induced ISG56 expression (data not shown). By contrast, no induction of ISG56 was observed after treatment with the TLR9 agonist ODN1826 or the TLR7 agonist ssRNA40 (Fig. 2G and data not shown). TLR2, 3, 4 have been reported to be involved in recognition of *Chlamydia* species [7], [29], [30], [30,31], and therefore we inhibited LPS/TLR4 action using polymyxin B and also the endosomal TLRs (TLR3, 7, 8, 9) using chloroquine. Inhibition of LPS activity did not affect the ISG response to *C. pneumoniae*, but treatment of the cells with chloroquine strongly inhinited the ability of the bacteria to induce ISG56 expression (Fig. 2H).

Recently it has been reported that RNA polymerase III transcribes AT-rich DNA in the cytoplasm, thus generating RNA-species recognized by RLRs (refs). To examine if the ISG response during *C. pneumoniae* infection was triggered through this pathway, we inhibited RNA polymerase III with ML-60218 (30 µM) and infected with the bacteria. However, this treatment did not affect *C. pneumoniae*-induced ISG56 expression (data not shown), thus suggesting that MAVS signaling during *C. pneumoniae* infection is not activated by RNA polymerase III driven transcription of AT-rich regions in the bacterial genome. Collectively, *C. pneumoniae* infection stimulates expression of ISGs via recognition by endosomal TLRs and cytoplasmic RLRs.

### Induction of ISGs by *Chlamydia pneumoniae* is dependent on TBK1/IKKε, bacterial replication and the type III secretion system

**Figure 3:**
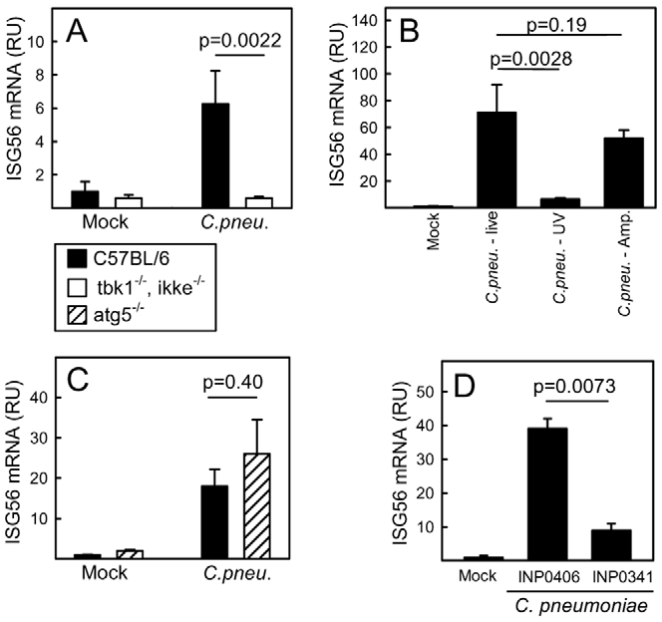
Induction of ISGs by *Chlamydia pneumoniae* is dependent on TBK1/IKKε, bacterial replication and the T3SS. (A, C) WT, tbk1^−/−^ ikke^−/−^ and atg5^−/−^ MEFs were infected with *C. pneumoniae*, MOI 5, for 36 h before harvest of total RNA. (B) WT MEFs were infected with live or UV-inactivated *C. pneumoniae* (CFU/cell 5) in the presence and absence of ampicillin (20 µg/ml) as indicated. (D) The cells were treated with INP0341 (60 µM), INP0406 (60 µM) or DMSO (vehicle) and infected with *C. pneumoniae*, MOI 5. Total RNA was harvested 36 hrs post-infection. (A–D) Levels of ISG56 mRNA were determined by real-time PCR. The measured ISG56 mRNA levels were normalized to β-actin, and data are presented as means of triplicate cultures +/− st.dev. Similar results were obtained in 2–3 independent experiments. RU, relative units.

The observed essential role for intracellular nucleotide-sensing PRRs for induction of ISGs by *C. pneumonia* prompted us to examine the potential role for the IRF-3 kinases TBK1 and IKKε, which are involved in signaling from most of these PRRs. As expected activation of the ISG response during *C. pneumonia* infection was dependent on these kinases (Fig. 3A).

The observed delayed kinetics of ISG expression relative to viral infections suggested that bacterial replication was necessary for induction of the response. To test this, we inactivated the bacteria with UV treatment and examined for induction of ISG56. As shown in Fig. 3B, inhibition of bacterial replication prevented induction of ISG56 expression.

The requirement for both endosomal TLRs, cytosolic RLRs and bacterial replication led us to hypothesize that interactions between the bacterial vacuole and the host cytoplasm are important for innate immune recognition and that cytoplasmic PAMPs are delivered to endosomes through autophagy. The potential role for autophagy was examined by comparing atg5+/− and atg5−/− cells with respect to *C. pneumoniae*-induced expression of ISG56 and showed that a deficient autophagy machinery did not impact on the IFN response evoked by *C. pneumoniae* (Fig. 3C). By contrast, inhibition of the type III secretion system using the inhibitor INP0341 largely prevented induction of ISG56 expression whereas the control compound INP0406 had no effect (Fig. 3D). Importantly, ampicillin, which halts the Chlamydia replication cycle before the conversion of RBs in to EBs but leaves interaction with the host cytosol unaffected [29], did not interfere with ISG56 expression. Thus, stimulation of ISG expression by *C. pneumonia* is dependent on bacterial replication, signaling through TBK1/IKKε and the T3SS.

## Discussion

*C. pneumoniae* is an obligate intracellular gram-negative bacteria responsible for about 10% of community-acquired pneumonias. *C. pneumoniae* has a 2-stage life-cycle involving infectious EB and metabolically active RB [3]. The host cytokine response to *Chlamydia* infection is involved in both protection and pathogenesis of the disease, with e.g. IFN-γ exerting bactericidal activities and interleukin 1 contributing to tissue damage [6]. Type I IFNs, which were originally believed primarily to participate in the host response to viral infections, have recently emerged as important players in host-defense and pathogenesis of bacterial infections. However, unlike the protective role of type I IFNs in antiviral defense, this class of cytokines has been reported to participate in development of disease during several bacterial infections [19]–[22]. For instance, type I IFNs enhance susceptibility of mice to genital and lung infection with *Chlamydia muridarum* through a mechanism involving inhibition of the CD4 T cell response and enhancement of macrophage apoptosis [19], [32]. Despite the role of type I IFN in the pathogenesis of *Chlamydia* infection, it remains unresolved which PRRs are responsible for induction of the IFN response during infection with *Chlamydia*. In this work we demonstrate that *C. pneumoniae* induces expression of ISGs with a delayed kinetics relative to viruses, and that this is dependent on bacterial replication, innate immune recognition by intracellular nucleotide-sensing PRRs and signaling through TBK1/IKKε. Finally, we report that induction of the ISG response is dependent on bacteria-host interaction through the T3SS.

The finding that *C. pneumoniae*-induced expression of ISGs is dependent on replication and occurs with a delayed kinetics relative to viruses suggests that PAMPs produced late during the bacterial life cycle (or exposed to PRRs only at late time points) are responsible for induction of the IFN response. Since the conversion of EBs to RBs occurs between 8 and 10 h after infection, it is tempting to speculate that the Chlamydial ISG-inducing nucleotide PAMP is produced or accessible only when RBs are actively dividing.

We found an essential role for the T3SS in induction of the ISG response. This is in agreement with a recent report that *C. muridarum* induces expression of a subset of inflammatory cytokines in a manner dependent on the T3SS [17]. The *Salmonella enterica* Typhimurium T3SS has also been reported to stimulate innate immune responses, but in that case it was reported to occur directly via bacterial effector proteins independent of any known innate immune receptors [33]. TLRs are believed to interact with the cytoplasm through autophagy [34]. However, abrogation of autophagy by deletion of the atg5 gene did not affect the ability of *C. pneumoniae* to stimulate ISG expression, suggesting other mechanisms to be involved. Others have reported that the TLR adaptor MyD88 localizes to the inclusions during *Chlamydia trachomatis* infections, and there may therefore be a more direct interaction between the Chlamydia inclusion and endosomal TLRs [29]. Therefore, based on our findings, we propose that active secretion of specific molecules by the T3SS during the RB stage of the bacterial life cycle either leads to exposure of PAMPs or amplifies host cell signaling, thus augmenting the ISG response. As to the first possibility, there is no direct evidence of nucleotides being translocated across T3SS. However, one recent work reported that synthetic RNA amplified the IFN-β response induced by *Yersinia pseudotuberculosis* and this was dependent on the T3SS [35]. Concerning the later possibility, this would be parallel to the recent finding that *C. trachomatis* infection stimulates production of reactive oxygen species through the T3SS, which leads to activation of the inflammasome and production of bioactive IL-1β [18].

Bacteria are recognized through all known classes of PRRs, and specifically for Chlamydia it is known that TLR2, TLR4, NOD1, NOD2, NLRP3 and possibly also TLR3 play a role in innate recognition of the bacteria [8], [9], [18], [29]–[31], [36]. In this work we found that endosomal TLRs (which include the nucleotide-sensing TLR3, 7, and 9) and cytoplasmic RLRs (which include the RNA-sensing RIG-I and MDA5) were essential for induction of the ISG response by *C. pneumonia*, and that the MEFs induced ISG56 expression after stimulation with agonists for TLR3, RIG-I, and MDA5 but not after stimulation with agonists for TLR7 and 9. Therefore, the ISG response to *C. pneumoniae* is likely to be mediated by intracellular RNA-sensing PRRs. This is in contrast to most reports on bacterial induction of the IFN response, which is mediated primarily by cytoplasmic DNA-sensing PRRs [12], [14], [25], [26]. However, one recent study demonstrated recognition of group B streptococcus in TLR7 in conventional dendritic cells as a central mechanism of type I IFN induction [27]. Previously we found that purified DNA from different bacteria all stimulated TLR9, but only a subset of the live bacteria stimulated this PRR [37]. This suggests that the nature of the interactions between host cells and the bacterial life cycle, rather than the intrinsic immunostimulatory potential of bacterial nucleotides, are responsible for which PRRs that mediate the innate immune response to infection.

In conclusion, we report that activation of the type I IFN response during *C. pneumoniae* infection is mediated by intracellular nucleotide-sensing PRRs, which operate through a mechanism dependent on the bacterial T3SS. Thus, specific strategies to inhibit the chlamydial T3SS may be used to limit the detrimental effects of the type I IFN system in the host response to Chlamydia infection, and hence to limit the pathology of infections with this intracellular bacterial pathogen.
